# Functional regulation of Zfp36l1 and Zfp36l2 in response to lipopolysaccharide in mouse RAW264.7 macrophages

**DOI:** 10.1186/s12950-015-0088-x

**Published:** 2015-07-16

**Authors:** Kuan-Ting Wang, Hsin-Hui Wang, Yan-Yun Wu, Yu-Lun Su, Pei-Yu Chiang, Nien-Yi Lin, Shun-Chang Wang, Geen-Dong Chang, Ching-Jin Chang

**Affiliations:** Graduate Institute of Biochemical Sciences, College of Life Science, National Taiwan University, No.1 Sec. 4 Roosevelt Road, Taipei, 10617 Taiwan; Department of Pediatrics, Division of Pediatric Immunology and Nephrology, Taipei Veterans General Hospital, No.201, Sec. 2, Shipai Road, Beitou District, Taipei, 112 Taiwan; Department of Pediatrics, Faculty of Medicine, School of Medicine, and Institute of Emergency and Critical Care Medicine, School of Medicine, National Yang-Ming University, No.155, Sec.2, Linong Street, Beitou District, Taipei, 112 Taiwan; Institute of Biological Chemistry, Academia Sinica, No.128, Sec.2, Academia Road, Nankang, Taipei, 11529 Taiwan

**Keywords:** Tristetraprolin, Zfp36l1, Zfp36l2, Mkp-1, p38 MAPK, Macrophage

## Abstract

**Background:**

The tristetraprolin (TTP) family of mRNA-binding proteins contains three major members, Ttp, Zfp36l1, and Zfp36l2. Ttp down-regulates the stability of AU-rich element–containing mRNAs and functions as an anti-inflammation regulator.

**Methods:**

To examine whether other TTP family proteins also play roles in the inflammatory response, their expression profiles and the possible mRNA targets were determined in the knockdown cells.

**Results:**

*Ttp* mRNA and protein were highly induced by lipopolysaccharide (LPS), whereas *Zfp36l1* and *Zfp36l2* mRNAs were down-regulated and their proteins were phosphorylated during early lipopolysaccharide stimulation. Biochemical and functional analyses exhibited that the decrease of *Zfp36l2* mRNA was cross-regulated by Ttp. Knockdown of Zfp36l1 and Zfp36l2 increased the basal level of *Mkp-1* mRNAs by prolonging its half-life. Increasing the expression of Mkp-1 inhibited the activation of p38 MAPK under lipopolysaccharide stimulation and down-regulated *Tnfα*, and *Ttp* mRNA. In addition, hyper-phosphorylation of Zfp36l1 might stabilize *Mkp-1* expression by forming a complex with the adapter protein 14-3-3 and decreasing the interaction with deadenylase Caf1a.

**Conclusions:**

Our findings imply that the expression and phosphorylation of Zfp36l1 and Zfp36l2 may modulate the basal level of *Mkp-*1 mRNA to control p38 MAPK activity during lipopolysaccharide stimulation, which would affect the inflammatory mediators production. Zfp36l1 and Zfp36l2 are important regulators of the innate immune response.

**Electronic supplementary material:**

The online version of this article (doi:10.1186/s12950-015-0088-x) contains supplementary material, which is available to authorized users.

## Background

Activated macrophages produce the classical pro-inflammatory cytokines to initiate and maintain inflammation. The activities of mitogen-activated protein kinases (MAPKs) including ERK, JNK, and p38 are necessary for initiation of the innate immune response [1]. Both the activation of transcription factors by phosphorylation and the stabilization of AU-rich element (ARE)-containing mRNAs can help cells produce pro-inflammatory mediators or cytokines [2–4]. Some negative regulators such as MAPK phosphatase-1 (Mkp-1, also named Dusp1, dual specific phosphatase 1) and tristetraprolin (Ttp) can help cells return to the resting state [5, 6]. MAPKs are inactivated by Mkp-1 through dephosphorylation [7]. *Mkp-1*-deficient mice are highly susceptible to endotoxic shock, which is associated with enhanced production of serum cytokines and chemokines [8–11]. In *Ttp*-knockout mice, many inflammation syndromes such as dermatitis, cachexia, spontaneous arthritis, and neutrophilia are observed [12] because of overproduction of the pro-inflammatory cytokine tumor necrosis factor-α (Tnfα) through its prolonged mRNA half-life [13]. Ttp (also called TIS11, NUP475, Zfp-36, and G0S24) specifically binds to the ARE in the 3′UTR of *Tnfα* mRNA. The ARE is a *cis*-acting RNA element that is usually located in the 3′UTR of short-lived mRNAs encoded by many inflammation- and cancer-associated genes [6]. TTP recognizes the ARE via its tandem zinc finger domain, which contains two Cys-Cys-Cys-His (C3H) zinc-binding motifs, and causes its target mRNA to be deadenylated and rapidly degraded [14–16].

The other TTP family proteins are Zfp36l1 (also called TIS11b, cMG1, BRF1, ERF1, or Berg36) [17], Zfp36l2 (also called TIS11d, ERF2, or BRF2) [18] and Zfp36l3 [19]. These members contain conserved tandem zinc finger domains that show the same ability as Ttp to destabilize ARE-containing mRNAs [15, 20]. Their RNA expression levels vary among human tissues [21]. *Zfp36l1*-deficient mice develop failure of chorioallantoic fusion, and embryos die *in utero* [22]. Female mice containing a deletion of the first exon of *Zfp36l2* are completely infertile [23]. *Zfp36l2*-knockout mice exhibit a defect in hematopoiesis [24]. *Zfp36l3* is rodent-specific [19]; it is only expressed in the placenta and extraembryonic tissues of mice.

Ttp mRNA and protein are induced by lipopolysaccharide (LPS) [25]; however, Zfp36l1 and Zfp36l2 proteins have not been adequately characterized in LPS-stimulated macrophages. To explore the functional roles of Zfp36l1 and Zfp36l2 in inflammatory response, we examined their expression profiles and knocked down either Zfp36l1 or Zfp36l2 to find their possible mRNA targets in mouse RAW264.7 cells. We observed that Zfp36l1 and Zfp36l2 would modulate *Mkp-1* mRNA expression in resting macrophages, which inhibited p38 activation and *Tnfα* induction in response to LPS.

## Methods

### Plasmid constructs

The Flag-tagged mouse Ttp, Zfp36l1, and Zfp36l2 expression plasmids, the construct encoding the GST fusion with mouse 14-3-3**ζ**, and pCMV-Tag2C-luciferase-Mkp-1 3′UTR were constructed as described [26]. The sequence of putative AREs of MKP-1 3’UTR was indicated previously [27]. T7 promoter containing Zfp36l1 and Zfp36l2 partial 3’UTR were PCR cloning by using primers, 5’-TAATACGACTCACTATAGGGGTTGCTTATCACTGCACATC-3’ and 5’-AAACTGCAAATAGTCGTTAC-3’ for Zfp36l1, 5’-TAATACGACTCACTATAGGGCACCACTGCACCACAACTC-3’ and 5’-AAGCATGGTTTCTTCATGCG-3’ for *Zfp36l2*. After sequences confirmed, these two fragments were cloned into 3’ of luciferase gene in pCMV-Tag2C-luciferase plasmid (Stratagene, La Jolla, CA).

### Cell culture

RAW264.7 cells were grown in Dulbecco’s modified Eagle medium (Gibco, Grand Island, NY) containing 1.5 g · L^–1^ sodium bicarbonate and supplemented with 10 % fetal bovine serum (HyClone, Logan, UT) and 2 mM l-glutamine (Gibco). HEK293T cells were grown in Dulbecco’s modified Eagle medium (Gibco) containing 3.7 g · L^–1^ sodium bicarbonate and supplemented with 10 % fetal bovine serum (Gibco). 100 ng · mL^–1^ LPS and 20 μM of BAY11-7082 were used (Sigma-Aldrich, St Louis, MO). Both RAW264.7 and HEK293T cells were cultured at 37 °C in a humidified incubator with 5 % CO_2_.

### Preparation of whole-cell extracts and cytoplasmic/nuclear extracts

Confluent RAW264.7 cells in a 10-cm dish were washed once with phosphate-buffered saline (PBS) and then harvested. To prepare whole-cell extracts, the harvested cells (5 × 10^6^) were lysed in 400 μL of whole-cell extract buffer (25 mM HEPES pH 7.7, 1.5 mM MgCl_2_, 0.2 mM EDTA, 0.5 mM dithiothreitol (DTT), 0.1 % v/v NP-40, 0.3 M NaCl, protease inhibitor cocktail (Sigma-Aldrich) and phosphatase inhibitor containing 0.01 M β-glycerol phosphate, 0.1 mM Na_2_MoO_4_, 0.1 mM Na_3_VO_4_ pH 10, 0.01 M NaF). The cell lysates were shaken at 4 °C for 30 min and then centrifuged for 5 min at 13,000 rpm, 4 °C. The supernatant was collected as a whole-cell extract. To prepare cytoplasmic and nuclear extracts, harvested cells were lysed in 400 μL hypotonic buffer (10 mM HEPES pH 7.5, 10 mM potassium acetate, 2.5 mM DTT, 0.05 % NP-40, protease inhibitor, and phosphatase inhibitor). The cell lysates were shaken at 4 °C for 30 min and then centrifuged for 30 s at 9000 rpm, 4 °C. Each supernatant was collected as a cytosolic extract, and each nuclear pellet was washed once with hypotonic buffer and then resuspended in 50 μL of buffer C (20 mM HEPES pH 7.9, 400 mM NaCl, 1 mM EDTA, 1 mM EGTA, 1 mM DTT, protease inhibitor, and phosphatase inhibitor). The nuclear suspension was shaken at 4 °C for 30 min and then centrifuged for 5 min at 13,000 rpm, 4 °C. Each supernatant was collected as a nuclear extract. For phosphatase treatment, 100 μg of cytoplasmic extract was incubated with 1 μL of calf intestinal phosphatase (New England Biolabs, Ipswich, MA) at 37 °C for 30 min.

### Western blot analysis and antibodies

Four fold of SDS-PAGE sample buffer (200 mM Tris pH 6.8, 8 % SDS, 0.4 % bromophenol blue, 40 % glycerol, 400 mM β-mercaptoethanol) was added to the sample to a final concentration of 1 fold and then heated at 100 °C for 5 min. Proteins were separated on 10 % polyacrylamide gels and transferred onto a 0.45 μm-pore-size polyvinylidene difluoride membrane (Millipore, Billerica, MA) for western blotting. The membrane was incubated for 1 h at room temperature with an antibody against any of the following proteins: BRF1/2, phosphorylated p38 (p-p38) MAPK T180/Y182, p-p44/42 MAPK (all from Cell Signaling), hnRNPC1/C2, MKP-1, ERK1, JNK1 (all from Santa Cruz Biotechnology), total-p38, p-JNK, Flag M2 (all from Sigma-Aldrich), Ttp, Zfp36l1, Zfp36l2, and β-tubulin [26]. After washing with PBST (PBS containing 0.1 % (v/v) Tween 20) for an appropriate time, the membrane was incubated for 1 h at room temperature with a horseradish peroxidase–conjugated secondary antibody: goat anti-rabbit IgG (KPL, Gaithersburg, ML), goat anti-mouse IgG (KPL), or rabbit anti-goat (Sigma-Aldrich). Western Lightning enhanced chemiluminescence substrate (Perkin Elmer, Norwalk, CT) was used for detection.

### RNA extraction, reverse transcription and real-time PCR

Total RNA was isolated with TRIzol reagent (Invitrogen, Carlsbad, CA) according to the manufacturer's suggestions. Cells (1 × 10^6^) in a six-well plastic culture plate were washed once with PBS and directly lysed in a well with 1 mL TRIzol. RNA was used for reverse transcription as described [26]. RNA was quantified with the Applied Biosystems 7300 Real-Time PCR system (Applied Biosystems, Foster City, CA) in a volume of 20 μL, containing 10 μL FastStart Universal SYBR Green Master (Roche, Mannheim, Germany), 4 μL of 10-fold diluted cDNA, 5.6 μL diethylpyrocarbonate-treated H_2_O, and 0.4 μL of 5–20 μM forward and reverse primer: 5′-TAGACTCCATCAAGGATGCTGG-3′ and 5′-GCAGCTTGGAGAGGTGGTGAT-3′ for *Mkp-1*; 5′-GACCCTCACACTCAGATCATCTTCT-3′ and 5′-CCTCCACTTGGTGGTTTGCT-3′ for *Tnfα*; 5′-GGATCTCTCTGCCATCTACGA-3′ and 5′-CAGTCAGGCGAGAGGTGAC-3′ for *Ttp*; 5′-CTGAAGACCTTAGGGCAGAT-3′ and 5′-AAGGAATGGGTCCAGACATAC-3′ for *Ccl2*; 5′-TGTCAGCCACTGCCTTGGTA-3′ and 5′-CAGGATCTGGTCCGCTAGCT-3′ for *Icam1*; 5’-TGAGCGAAGTTTTATGCAAGGG-3’ and 5’-GCTGGGCAGAGTGACCGAG-3’ for *Zfp36l1*, 5’-GATGTCGACTTGTTGTGCAAGACG-3’ and 5’-GCGTCCCTACCGCCTTCT-3’ for *Zfp36l2*, 5′-TCCTTCCTGGGCATGGAGTC-3′ and 5′-ACTCATCATACTCCTGCTTG-3′ for *β-actin*. The data were normalized with β-actin according to the 2^–ΔΔCt^ relative quantitation method in the manufacturer's manual.

### RNA pull-down assay

Cytoplasmic extracts from LPS-stimulated RAW264.7 cells were collected as described above. Potassium acetate was adjusted to 90 mM, and 0.1 U · μL^-1^ RNasin (Promega, Madison, WI) and 20 μg · μL^-1^ yeast tRNA were added to each lysate. To prevent non-specific binding, heparin-agarose (Sigma-Aldrich) was incubated with each lysate for 15 min at 4 °C and then centrifuged for 1 min at 8000 rpm, 4 °C. Each supernatant was further cleaned with streptavidin-Sepharose (8 μL; Invitrogen) for 1 h at 4 °C and then centrifuged for 1 min at 8000 rpm, 4 °C. *Mkp-1* 3’UTR cloned in T7 promoter-containing plasmid or T7 promoter-containing *Zfp36l1* 3’UTR or *Zfp36l2* 3’UTR DNA fragment was used as a template and transcribed into RNA in the presence of biotin-CTP by using the T7-MEGA shortscript, High Yield Transcription kit (Ambion, Grand Island, NY) and then incubated with above supernatants for 1 h at 4 °C. Biotin-labeled 18S RNA was a negative control. Next, streptavidin-Sepharose (8 μL) was added to the pulled-down biotinylated RNA complex for 2 h at 4 °C. The pulled-down complexes were washed four times with binding buffer (hypotonic buffer containing 90 mM potassium acetate). Finally, the RNA complexes were separated by SDS-PAGE (10 % acrylamide) and detected by western blotting.

### Dual luciferase reporter assay

HEK293T cells were seeded in a six-well plastic culture plate and transfected using calcium phosphate precipitation with different plasmids (containing 0.25 μg *Renilla* luciferase expression vector as a control of transfection rate) at 30 % confluency. At 24 h post-transfection, the cells (5 × 10^5^) were harvested and lysed in 50 μL of passive lysis buffer (Promega). The samples were shaken for 30 min at 4 °C and centrifuged for 5 min at 13,000 rpm, 4 °C. The supernatants were collected for Dual-Luciferase reporter assay (Promega). The firefly luciferase activity was normalized with the *Renilla* luciferase activity. All the experiments were carried out in duplicate and repeated for three times.

### Lentivirus knockdown

Lentivirus vectors encoding shRNA targeted to mouse *Zfp36l1*, *Zfp36l2*, and control *Luciferase* were purchased from the National RNAi core facility (Academia Sinica) and used in knockdown studies as described [26]. HEK293T cells (1 × 10^6^) were seeded in a 10-cm dish for transfection (calcium phosphate precipitation method) of virus production vectors, 14 μg of CMV ΔR8.9.1, 2 μg of pMD.G, and 14 μg of specific shRNA sequence–bearing pLKO.1 plasmids. At 8 h post-transfection, the culture medium was replaced with fresh medium for RAW264.7 cells. Virus-containing medium was collected 24 and 48 h later for primary-infection and super-infection of RAW264.7 cells. Virus-containing medium was replaced with fresh medium for RAW264.7 cells 24 h after super-infection. To generate stable knockdown clones, puromycin (3 μg · mL^-1^) was added and Green fluorescent protein signal served as a selection marker. After puromycin selection for one week, cells were harvested and analyzed by western blotting to determine knockdown efficiency.

### GST fusion protein production and GST pull-down assay

Glutathione-Sepharose 4B beads (approximately 8 μl, GE Healthcare, Piscatway, NJ) were incubated with bacterially expressed GST or GST–14-3-3 proteins in PBS containing 1 % (v/v) Triton X-100 on a rotary shaker for 20 min at room temperature. After washing three times with the same buffer, the beads were combined with 500 μg of each cell lysate from LPS-stimulated RAW264.7 cells in a final volume of 200 μl of buffer containing 20 mM HEPES, pH 7.9, 100 mM NaCl, 2.5 mM MgCl_2_, 0.1 mM EDTA, 0.05 % NP-40, and 1 % Triton X-100, along with 1 mM DTT, and 1 mM phenylmethylsulfonyl fluoride, on a rotary shaker. The mixtures were incubated at 4 °C for 2 h, and then the beads were washed four times with the same buffer lacking DTT and phenylmethylsulfonyl fluoride but containing 200 mM NaCl and washed once with 50 mM Tris, pH 6.8. Bound proteins were eluted by boiling in SDS-PAGE sample buffer and analyzed by immunoblotting.

### Statistical analysis

All of the results are presented as the mean ± SD of at least three independent experiments. The statistically significant values were calculated by one-tailed Student's *t*-test. One asterisk indicates *P*-value < 0.05, and two asterisks indicate *P*-value < 0.01. ns indicates non-significance.

## Results

### Constitutive expression and phosphorylation of Zfp36l1 and Zfp36l2 during early LPS stimulation in RAW264.7 cells

**Fig. 1:**
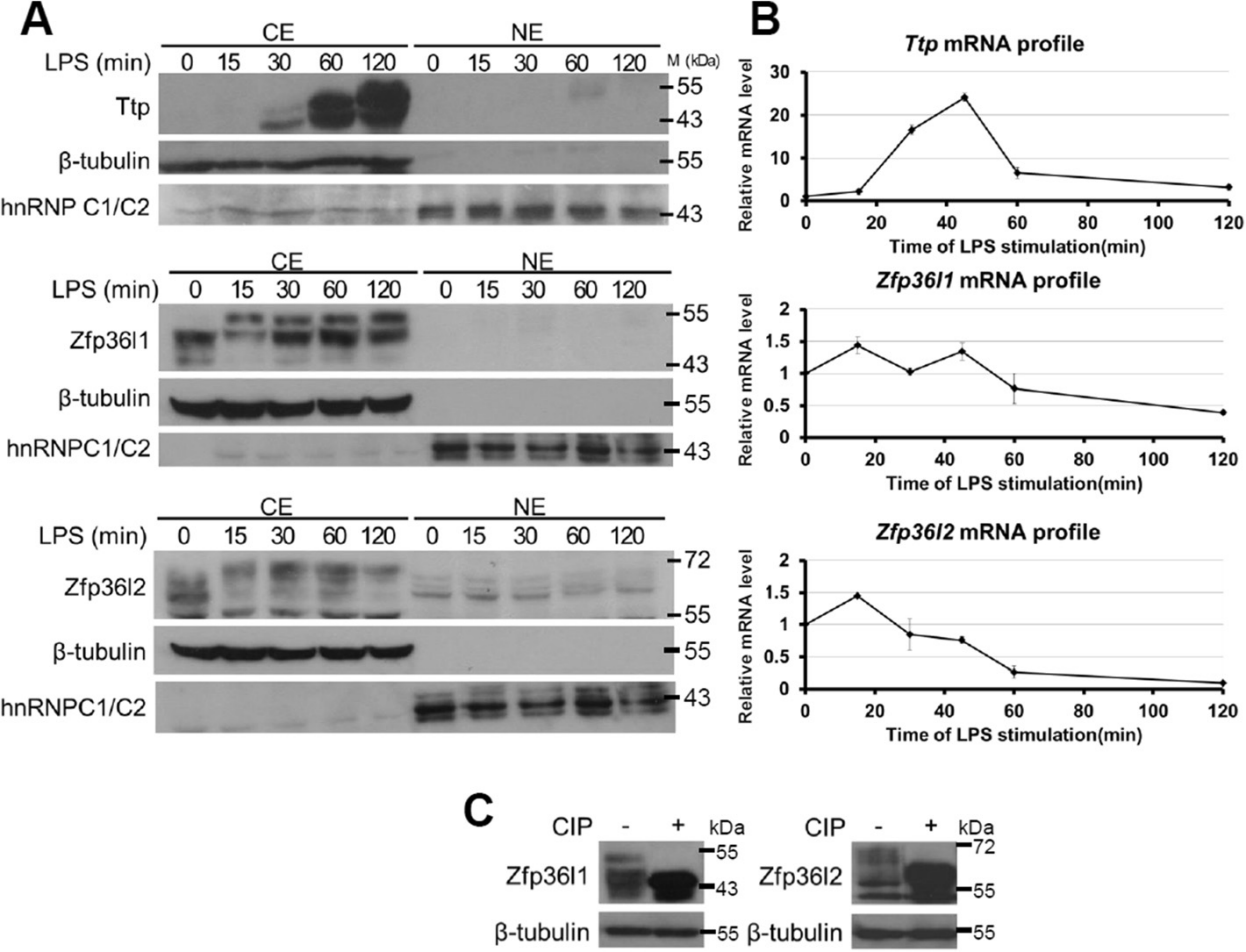
Consistent expression and phosphorylation of Zfp36l1 and Zfp36l2 during LPS stimulation in RAW264.7 cells. **a** Protein expression profiles of TTP family members in RAW264.7 cells stimulated with LPS for 0, 15, 30, 60, or 120 min. Cytoplasmic extracts (CE) and nuclear extracts (NE) were isolated for western blotting analysis using the indicated antibodies. β-tubulin was a loading control for the cytoplasmic extract, and hnRNPC1/C2 was a loading control for the nuclear extract. Positions of molecular size markers (M) are shown to the right. The blots were performed at least three times, and representative results from replicate experiments were presented. **b** mRNA expression profiles of TTP family members in RAW264.7 cells stimulated with LPS for 0, 15, 30, 60, or 120 min. RNA was isolated for quantitative PCR analysis to measure *Ttp, Zfp36l1, Zfp36l2* mRNA levels. The relative mRNA level was shown as mean ± SD of three independent samples normalized to β-actin mRNA level. **c** Cytosolic extracts from RAW264.7 cells stimulated with LPS for 120 min were treated with calf intestinal phosphatase for 30 min and then were subjected to SDS-PAGE for western blotting analysis

Ttp plays a key role in the innate immune response. Ttp mRNA and protein were highly induced by LPS (Fig. 1a, b). To investigate the roles of two other TTP family proteins, Zfp36l1 and Zfp36l2, in the inflammatory response, we first examined their RNA and protein expression profiles in LPS-stimulated RAW264.7 cells. Zfp36l1 and Zfp36l2 proteins were near consistently maintained in the cytoplasm during early LPS stimulation (Fig. 1a) although their RNA expression levels decreased (Fig. 1b). Multiple forms of Zfp36l1 and Zfp36l2 were detected by western blotting, and LPS treatment resulted in a shift in their bands (Fig. 1a). When cytoplasmic extracts from 120-min LPS-stimulated cells were treated with calf intestinal phosphatase for 30 min, the higher-migrating protein bands of Zfp36l1 and Zfp36l2 shifted back to their lower positions (Fig. 1c). These observations suggested that Zfp36l1 and Zfp36l2 proteins were maintained at a constant level and were phosphorylated under LPS stimulation. They might play functions in resting state and their activity might be regulated by protein phosphorylation.

### Down-regulation of *Zfp36l1* and *Zfp36l2* mRNA by Ttp during LPS-stimulation

**Fig. 2:**
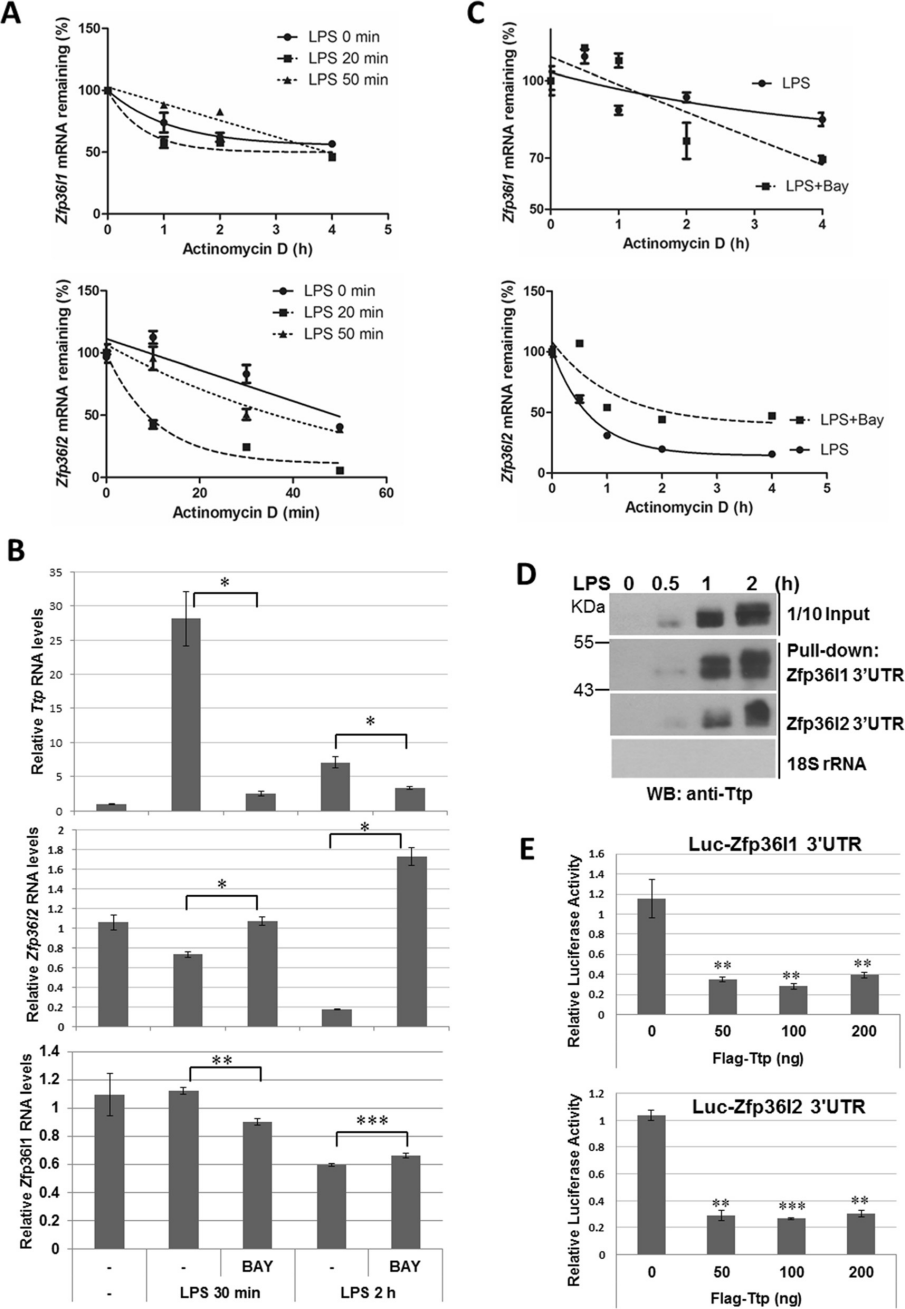
mRNA stability regulation of *Zfp36l2* by Ttp during LPS-stimulation. **a** mRNA half-life determination. RAW264.7 cells stimulated with LPS for 0, 20 and 50 min, and actinomycin D (10 μg · mL^-1^) was added for 0, 10, 30, and 50 min to stop transcription for *Zfp36l2* mRNA detection, and added for 0, 1, 2 and 4 h for *Zfp36l1* mRNA detection. RNAs was isolated for quantitative PCR by using primers of *β*-*actin*, *Zfp36l1* and *Zfp36l2*. The remaining *Zfp36l1* and *Zfp36l2* mRNA levels were shown after normalized with the level of *β*-*actin.* The mRNA half-lives were calculated by exponential regression: 5.2 h, 4.2 h, and 3.5 h for *Zfp36l1* at 0, 20 min, and 50 min of LPS treatment, respectively; 39 min, 13 min and 33 min for *Zfp36l2* at 0, 20 min, and 50 min of LPS treatment, respectively. **b** TTP family mRNA analysis under BAY treatment. RAW264.7 cells were pretreated with or without 20 μM of BAY for 0.5 h followed by adding 100 ng · mL^-1^ LPS for 0.5 and 2 h. Total RNAs were isolated for quantitative PCR. **c** BAY treatment stabilizes *Zfp36l2* mRNA. RAW264.7 cells pretreated with or without 20 μM of BAY for 0.5 h followed by adding 100 ng · mL^-1^ LPS for 20 min, and actinomycin D was added for 0, 0.5, 1, 2, and 4 h to stop transcription. RNAs was isolated for quantitative PCR and *Zfp36l1* and *Zfp36l2* mRNA half-lives were determined. **d** RNA pull-down analysis. Biotin labeled *Zfp36l1* 3’UTR, *Zfp36l2* 3’UTR and control 18S RNA were incubated with cytosolic extracts from RAW264.7 cells treated with LPS for 0, 0.5, 1 and 2 h, respectively. After extensive washes, the RNA-protein complexes were analyzed by western blotting with anti-Ttp. **e** Luciferase reporter analysis. 293 T cells were cotransfected with of 0.25 μg *Zfp36l1* 3’UTR- or *Zfp36l2* 3’UTR-containing luciferase reporter and different amounts of Flag-tagged Ttp expression plasmid. After normalized with internal control of Renilla luciferase activity, the relative firefly luciferase activity was shown. All experiments were independently repeated at least two times

We are interested in the molecular mechanism of down-regulation of *Zfp36l1* and *Zfp36l2* mRNA in response to LPS treatment. Their mRNA 3’UTR contains potential AREs (Additional file 1: Figure S1), we examined whether their mRNA half-life was regulated by LPS. As shown in Fig. 2a, the half-life of *Zfp36l1* mRNA was longer than that of *Zfp36l2* mRNA, which was 5.2 h for *Zfp36l1* and 39 min for *Zfp36l2*. The half-life of *Zfp36l2* mRNA was significantly shortened to 13 min at 20 min of LPS treatment, and then restored to 33 min at 50 min treatment. Although the half-lives of Zfp36l1 mRNA were also decreased upon LPS treatment, they still maintained to near 4 h (Fig. 2a). It was known that Ttp induced by LPS can destabilize ARE-containing mRNA [13, 25]. We have demonstrated that NF-κB signaling pathway was required for Ttp induction [28]. To examine whether Ttp expression plays roles in the down-regulation of *Zfp36l1* and *Zfp36l2* mRNA, we compared the mRNA expression of *Ttp, Zfp36l1*and *Zfp36l2* in the presence of the inhibitor (BAY) of NF-κB pathway. The pretreatment with BAY inhibited the mRNA level of *Ttp* in LPS-stimulation for 0.5 h and 2 h, which was consistent with the increases of *Zfp36l2* mRNA level (Fig. 2b), but no correlation with the levels of *Zfp36l1* mRNA. The increase of *Zfp36l2* mRNA under BAY treatment was due to the increase of mRNA half-life, whereas BAY treatment would shorten *Zfp36l1* mRNA half-life (Fig. 2c). Moreover, the biotin-labeled partial 3’UTR from *Zfp36l1* or *Zfp36l2* associates with Ttp proteins in RNA pull-down analysis (Fig. 2d), and the 3’UTR-mediated luciferase activity was down-regulated when cotransfection with Ttp expression vector (Fig. 2e). Although both *Zfp36l1* and *Zfp36l2* 3’UTR showed physical and functional interaction with ectopic expressive Ttp, only endogenous *Zfp36l2* mRNA was decreased by LPS-induced NF-κB signals. The results suggest that in addition to Ttp there are other factors involved in the regulation of *Zfp36l1* mRNA stability during LPS stimulation.

### Zfp36l1 and Zfp36l2 destabilize *Mkp-1* mRNA in resting RAW264.7 cells

**Fig. 3:**
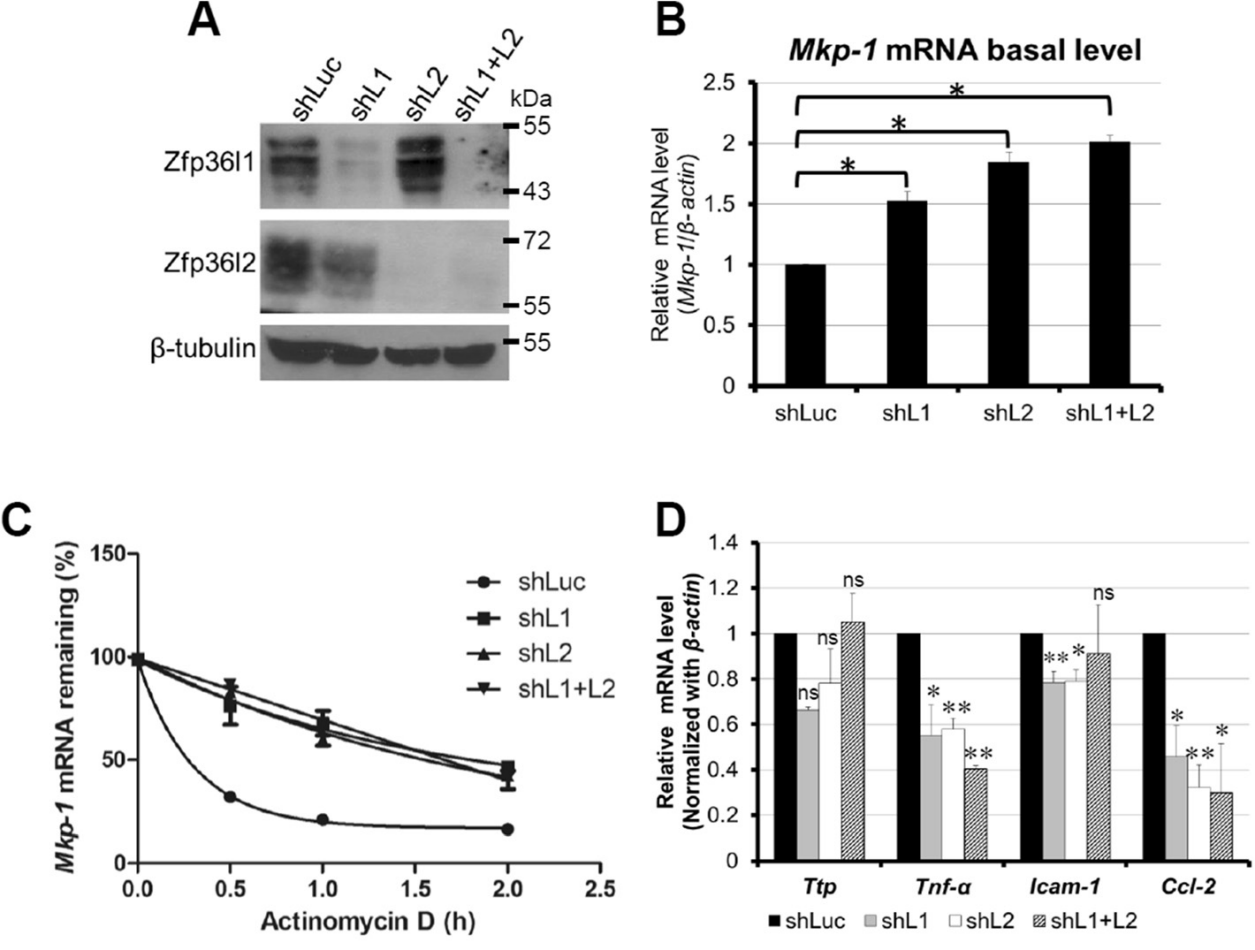
Zfp36l1 and Zfp36l2 destabilize *Mkp-1* mRNA in resting RAW264.7 cells. **a** shLuc, shL1, shL2, shL1 + L2 represent luciferase knockdown cells, Zfp36l1-knockdown cells, Zfp36l2-knockdown cells, and dual Zfp36l1- and Zfp36l2-knockdown cells, respectively. The upper two panels show the knockdown efficiency. β-tubulin was used as a loading control. Whole-cell extracts were collected for western blotting analysis using the indicated antibodies. **b** Basal levels of *Mkp-1* mRNA were detected by quantitative PCR in different knockdown cells. **c** Analysis of *Mkp-1* mRNA half-life in different knockdown cells. Actinomycin D (10 μg · mL^–1^) was added to stop transcription for 0, 0.5, 1 or 2 h. The remaining mRNA was detected by quantitative PCR. *Mkp-1* mRNA half-life was calculated by exponential regression, 19 min in control cells and 91, 96 and 95 min in Zfp36l1, Zfp36l2 and dual knockdown cells, respectively. **d** Basal levels of *Ttp*, *Tnfα*, *Icam-1*, and *Ccl-2* mRNAs were examined by quantitative PCR in different knockdown cells and normalized to the shLuc control. All of experiments were independently performed at least three times

Because Zfp36l1 and Zfp36l2 were expressed in control macrophages, we inferred that they play roles in controlling mRNA stability under resting conditions. The strategy to identify the mRNA targets of Zfp36l1 and Zfp36l2 was to knockdown Zfp36l1 and Zfp36l2 levels using lentivirus-carrying short hairpin RNAs (shRNAs) in RAW264.7 cells. The knockdown efficiency of shRNA specific to *Zfp36l1* and *Zfp36l2* was confirmed by western blotting (Fig. 3a). ARE-containing immediate early genes as well as inflammatory mediator genes such as *Ttp*, *Mkp-1*, *Tnfα*, *Ccl-2*, and *Icam-1* were candidate targets of Zfp36l1 and Zfp36l2 [29, 30]. Expression of these candidate RNAs was examined by real-time PCR in different knockdown cells, including Zfp36l1 knockdown, Zfp36l2 knockdown, and Zfp36l1/ Zfp36l2 dual-knockdown cells. We predicted that the mRNA targets of Zfp36l1 and Zfp36l2 would increase in knockdown cells because Zfp36l1 and Zfp36l2 function in mRNA destabilization. We found that *Mkp-1* mRNA was significantly increased in all knockdown cells (Fig. 3b). We determined the half-life of *Mkp-1* mRNA in the different knockdown cell types; the half-life increased from 19 min in control cells to near 100 min in knockdown cell types (Fig. 3c). In contrast, *Ttp*, *Tnfα*, *Ccl-2*, and *Icam-1* mRNA levels were decreased in the knockdown cells or slightly increased but no significance (Fig. 3d). These results suggested that both Zfp36l1 and Zfp36l2 down-regulate *Mkp-1* mRNA stability in resting macrophages but enhance the mRNA expression of *Tnfα* and *Ccl-2.*

### Zfp36l1 and Zfp36l2 interact with the *Mkp-1* 3′UTR and recruit the deadenylase Caf1a in RAW264.7 cells

**Fig. 4:**
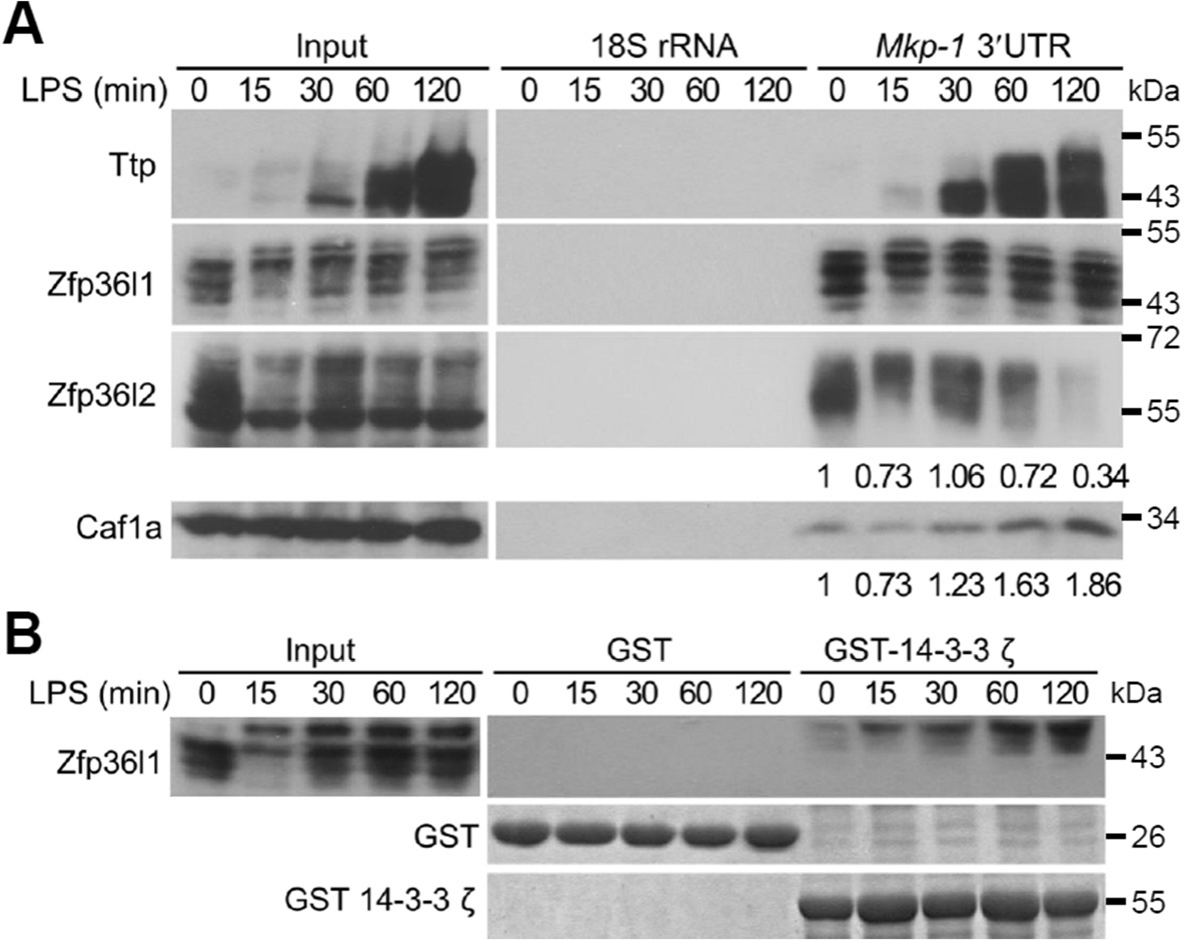
Zfp36l1 and Zfp36l2 interact with *Mkp-1* 3′UTR in RAW264.7 cells. **a** Interaction of TTP family proteins and *Mkp-1* mRNA. Biotinylated-*Mkp-1* 3′UTR fragments were incubated with cytosolic extracts from control RAW264.7 or LPS-treated cells for 15, 30, 60, or 120 min. The biotinylated RNAs and associated proteins were precipitated by streptavidin beads, separated by SDS-PAGE, and analyzed by western blotting with anti-Ttp, anti-Zfp36l1, anti-Zfp36l2, and anti-Caf1a as indicated. Biotinylated 18S rRNA was used as a negative control. The pull-down protein levels were quantified as indicated below the lanes. **b** Interaction of Zfp36l1 with 14-3-3. *E. coli*–expressed GST-14-3-3 ζ or GST was bound on glutathione Sepharose 4B. The beads were incubated with the cell lysates from LPS-stimulated RAW264.7 cells for 0, 15, 30, 60, or 120 min. The pulled-down protein complexes were separated by SDS-PAGE and analyzed by western blotting with anti-Zfp36l1. All of the experiments were performed at least three times, and representative data were displayed

We further explore the molecular mechanism underlying Zfp36l1 and Zfp36l2 regulated *Mkp-1* mRNA stability in resting macrophages. We have demonstrated that TTP family proteins including Ttp, Zfp36l1 and Zfp36l2 interacted with *Mkp-1* mRNA 3’UTR during differentiation of 3 T3-L1 preadipocytes [26, 27], and *Mkp-1* 3′UTR–derived luciferase activity was reduced when co-transfected with Zfp36l1 or Zfp36l2 expression plasmid in human embryonic kidney (HEK293T) cells [26]. The deadenylase CAF1 (Ccr4-associated factor) is recruited by TTP through interacting with NOT1 to destabilize target mRNAs [31–34]. Both Zfp36l1 and Zfp36l2 contain NOT1-binding domain [35]. A recent report showed that ZFP36L1 immunoprecipitated CAF1 (also named CNOT7) [36]. To understand how Zfp36l1 and Zfp36l2 regulate *Mkp-1* mRNA stability during LPS stimulation, an RNA pull-down assay was performed to examine the RNA-protein interaction. Biotinylated *Mkp-1* 3′UTR was incubated with cytosolic lysates from LPS-stimulated RAW264.7 cells. The ribonucleoprotein complexes were precipitated with streptavidin-Sepharose and then subjected to SDS-PAGE for western blotting with anti-Zfp36l1 and anti-Zfp36l2 (Fig. 4a). The interaction between Zfp36l1 and *Mkp-1* 3′UTR appeared to be constant during LPS stimulation, whereas the precipitated amount of Zfp36l2 by*Mkp-1* 3′UTR was varied from relative level 1 to 0.34 (Fig. 4a). Interestingly, the lowest level of precipitated Caf1a by *Mkp-1* 3′UTR was observed in LPS stimulation for 15 min, suggesting that *Mkp-1* mRNA would be stabilized after LPS stimulation.

It has been reported that the interaction between phosphorylated Ser92 and Ser203 of Zfp36l1 and the protein 14-3-3 can inhibit the mRNA decay activity of Zfp36l1 after insulin stimulation [37, 38]. Because LPS-induced Zfp36l1 phosphorylation did not affect its RNA-binding activity (Fig. 4a), a glutathione-S-transferase (GST) pull-down assay was performed to study whether Zfp36l1 that becomes hyper-phosphorylated during LPS stimulation interacts with 14-3-3. As shown in Fig. 4b, only hyper-phosphorylated Zfp36l1 formed a complex with 14-3-3. This complex might repress the mRNA destabilization function of Zfp36l1. No prominent Zfp36l2 was detected in this pull-down assay (data not shown). These results suggest that Zfp36l1 and Zfp36l2 bind to *Mkp-1* 3’UTR and may recruit RNA degradation complex in the resting macrophages. During LPS stimulation Zfp36l1 would be phosphorylated and sequestrated by 14-3-3 to decrease its mRNA destabilization effect, and the phosphorylated Zfp36l2 decreased its interaction with *Mkp-1* 3’UTR.

### Induction of *Mkp-1* mRNA early during LPS stimulation is post-transcriptionally modulated by Zfp36l1 and Zfp36l2

**Fig. 5:**
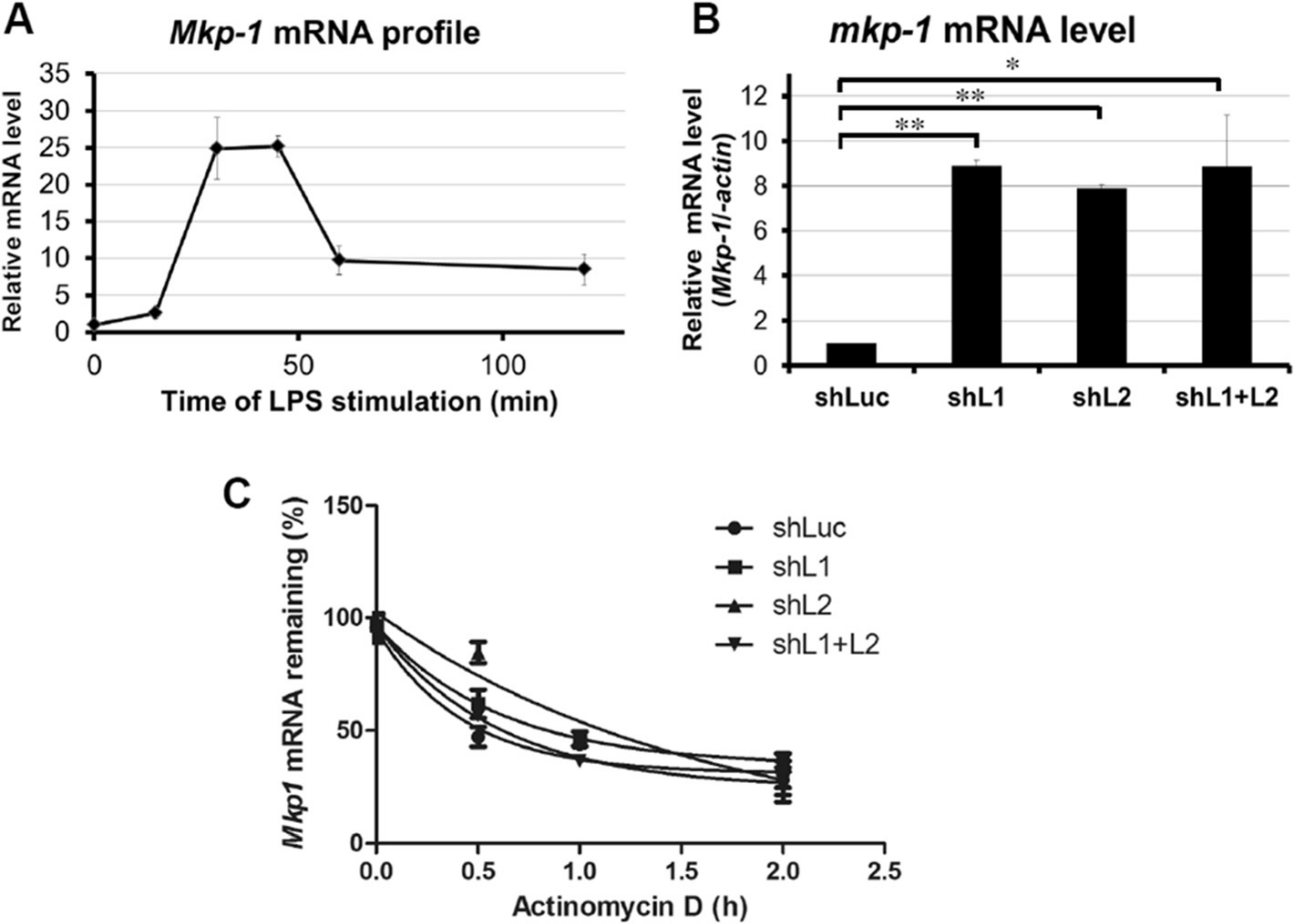
Induction of *Mkp-1* mRNA early during LPS stimulation is post-transcriptionally modulated by Zfp36l1 and Zfp36l2. **a** mRNA expression profile of *Mkp-1* in RAW264.7 cells stimulated with LPS for 0, 15, 30, 45, 60, or 120 min. **b** Levels of *Mkp-1* mRNA in different knockdown cells after LPS stimulation for 15 min. RNA was isolated and performed the real-time PCR analysis. **c** Analysis of *Mkp-1* mRNA half-life in different knockdown cells after LPS stimulation for 15 min. Actinomycin D (10 μg · mL^-1^) was added to stop transcription after 0, 10, or 20 min. The remaining mRNA was detected by quantitative PCR. *Mkp-1* mRNA half-life was calculated by exponential regression, 32 min, 55 min, 68 min, and 42 min in control, Zfp36l1, Zfp36l2, and dual-knockdown cells, respectively. All of experiments were carried out at least three times

To further investigate the regulation of *Mkp-1* mRNA during early LPS stimulation in RAW264.7 cells, we examined the expression of its mRNA (Fig. 5a). *Mkp-1* mRNA increased significantly after LPS stimulation from 15 to 30 min but decreased rapidly after 45 min. To verify the functions of Zfp36l1 and Zfp36l2 immediately following LPS treatment, we examined the level of *Mkp-1* mRNA in Zfp36l1- and Zfp36l2-knockdown cells after LPS stimulation for 15 min, in this time point Ttp protein was not induced significantly and the lowest level of brought-down Caf1a by *Mkp-1* 3’UTR was observed in Fig. 4a. The observed marked rise in the mRNA level in all knockdown cells compared with control knockdown cells implied that the decrease in Zfp36l1 and Zfp36l2 protein expression facilitated the expression of *Mkp-1* mRNA in response to LPS (Fig. 5b); furthermore, the relative *Mkp-1* mRNA half-life at LPS-stimulation 15 min in control, Zfp36l1, Zfp36l2, and dual-knockdown cells was 32 min, 55 min, 68 min, and 42 min, respectively (Fig. 5c). Our results indicate that knockdown of Zfp36l1, Zfp36l2, or both proteins also cause the increase of *Mkp-1* mRNA half-life at early LPS stimulation for 15 min like at the resting status. Moreover, *Mkp-1* mRNA at early LPS stimulation in control shLuc cells appeared more stable (half-life is 32 min) than which at the resting condition (half-life is 19 min) showed in Fig. 3c. It might be due to phosphorylation of Zfp36l1 and Zfp36l2 upon LPS stimulation leading to protein inactivation.

### p38 MAPK activity is regulated by Zfp36l1 and Zfp36l2 through Mkp-1 in LPS-stimulated RAW264.7 cells

**Fig. 6:**
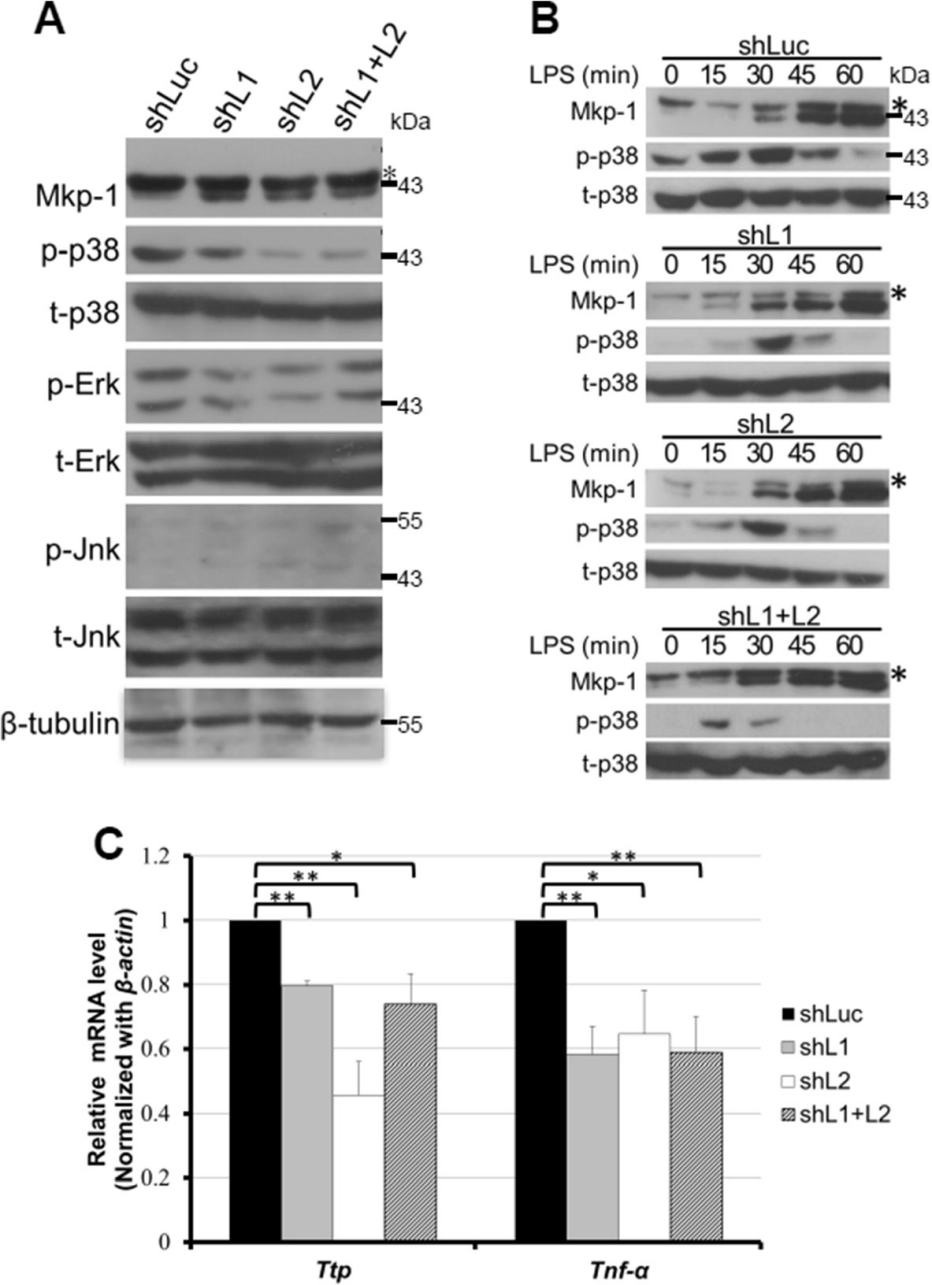
p38 MAPK activity is regulated by Zfp36l1 and Zfp36l2 through Mkp-1. **a** Basal activities of three MAPKs in Zfp36l1- and Zfp36l2-knockdown cells. Whole-cell extracts were isolated from RAW264.7 cells with Zfp36l1, Zfp36l2, or Zfp36l1/Zfp36l2 knockdown as indicated. Western blotting was performed to detect MAPKs and phospho-MAPKs. The asterisk indicates cross-reacted Mkp-2. **b** Mkp-1 expression and p38 activation profiles in different knockdown cells stimulated with LPS for 0, 15, 30, 45, or 60 min. Whole-cell extracts were collected and separated by SDS-PAGE, and analyzed by western blotting with anti-Mkp-1, anti-p-38, anti-p38, and anti-β-tubulin antibodies. The asterisk indicates cross-reacted Mkp-2. All of experiments were performed at least three times, and representative data were displayed. **c** The relative expression levels of *Ttp* and *Tnfα* mRNAs in different knockdown cells after LPS stimulation for 15 min. RNA was isolated and performed real-time PCR analysis

Because Mkp-1 has been reported to decrease p38 and Jnk activity as an anti-inflammation regulator [39], we further examined the three MAPK activities in Zfp36l1- and Zfp36l2-knockdown cells. Under resting conditions, p38 activity inversely correlated with Mkp-1 expression, but the activities of Erk and Jnk were unchanged (Fig. 6a). Following LPS stimulation, Mkp-1 expression was detected much earlier in knockdown cells than in control cells, and it down-regulated p38 activity (Fig. 6b). *Ttp* and *Tnfα* mRNA expression was reduced in all knockdown cells under LPS stimulation, presumably as a result of decreased p38 activity by Mkp-1 (Fig. 6c). We examined the mRNA expression of some of ARE-containing cytokines including *IL-1*, *IL-6* and *Ccl2* [40]. Their expression was decreased in Zfp36l1 and Zfp36l2 knockdown cells (Additional file 2: Figure S2). Collectively, our results suggest that both Zfp36l1 and Zfp36l2 are necessary for the initiation of the innate immune response in macrophages.

## Discussion

**Fig. 7:**
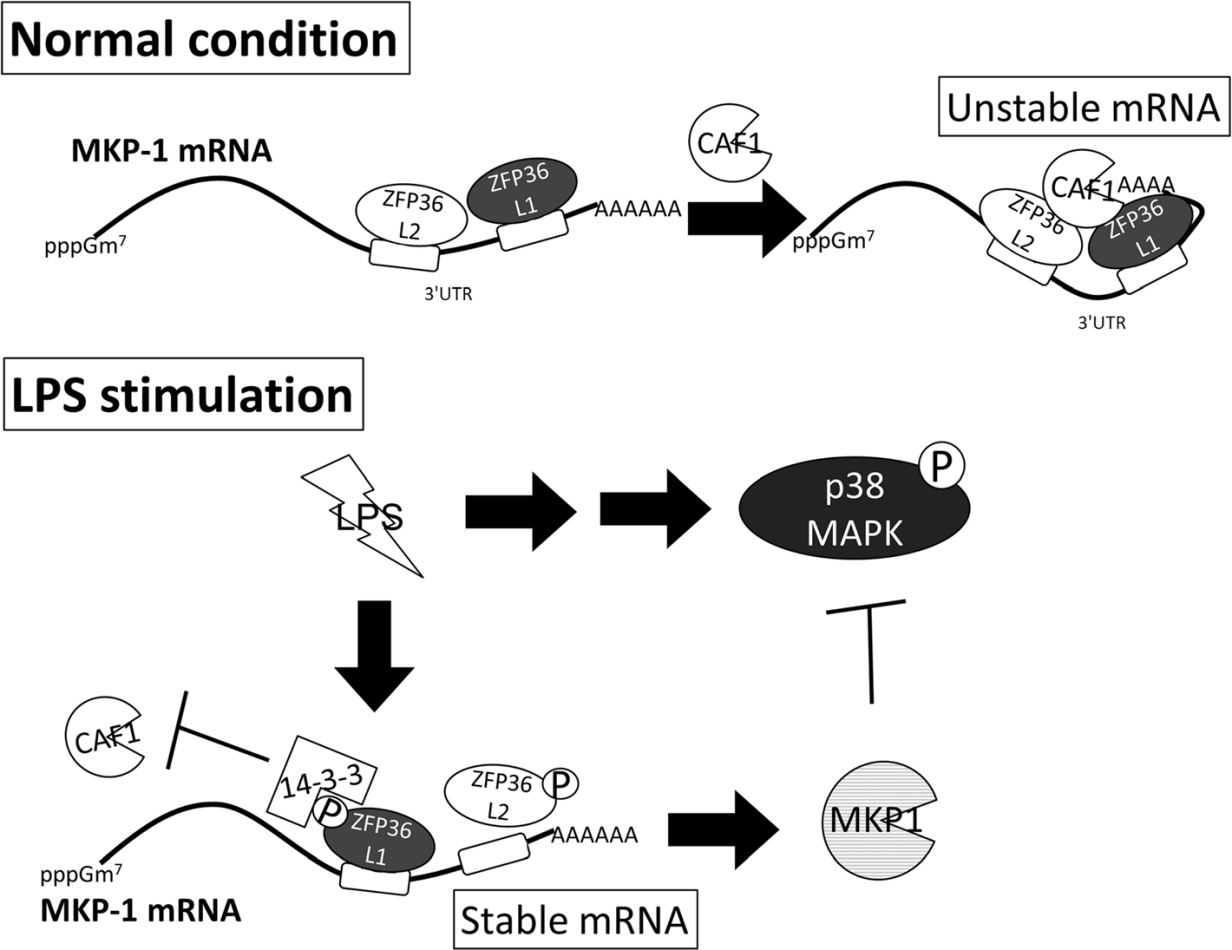
Hypothesized regulatory networks among Zfp36l1, Zfp36l2, Mkp-1, and p38 MAPK in RAW264.7 cells. Under resting conditions, Zfp36l1 and Zfp36l2 interact with *Mkp-1* mRNA 3’UTR and destabilize *it*, and the cells are sensitive to stimuli such as LPS under low *Mkp-1* expression. During early LPS stimulation for 15 min, Zfp36l1 and Zfp36l2 were phosphoprylated and resulted in the decrease of their mRNA destabilization function; so that *Mkp-1* mRNA and protein were induced to inactivate LPS-stimulated p38. pppGm^7^: 7-methylguanosine cap. P: phosphate group

Our data show that the levels of *Zfp36l1* and *Zfp36l2* mRNA decreased during LPS stimulation, and their proteins were consistently expressed and phosphorylated in response to LPS. Knockdown of Zfp36l1 and Zfp36l2 increased the expression of Mkp-1, and the activity of p38 MAPK was down-regulated under resting conditions. Thus, p38-mediated expression of *Ttp* and *Tnfα* mRNAs was repressed. According to these results, we propose the following model for the mechanism of Zfp36l1- and Zfp36l2-regulated *Mkp-1* expression in mouse macrophages (Fig. 7). Under a resting condition, Zfp36l1 and Zfp36l2 destabilize *Mkp-1* mRNA, and the cells are sensitive to stimuli such as LPS due to low *Mkp-1* expression. During transient LPS stimulation, *Mkp-1* mRNA is induced post-transcriptionally by hyper-phosphorylated Zfp36l1, which inhibits mRNA degradation, and by lower interaction with Zfp36l2.

There are two interesting features the expression profiles of endogenous TTP family genes during LPS stimulation in mouse macrophage RAW264.7 cells. One is that the protein expression profiles of these three TTP family members differed, and the second is that the mRNA and protein expression profiles of Zfp36l1 and Zfp36l2 were not correlated. Ttp was induced after LPS stimulation, and Zfp36l1 and Zfp36l2 were consistently expressed. This observation indicates that Zfp36l1 and Zfp36l2 may play important roles under resting conditions. The mRNA expression profiles of *Zfp36l1* and *Zfp36l2* were not correlated with their protein profiles after LPS stimulation (Fig. 1a, b). Similar observations regarding *Zfp36l1* and *Zfp36l2* mRNA expression profiles were reported by Liang et al. [41] and Cao et al. [42]. We had demonstrated that *Zfp36l2* mRNA was negatively regulated by Ttp (Fig. 2). This cross-regulation in TTP family proteins was also reported in yeast orthologs, Cth1 and Cth2 [43]. Furthermore, post-translational modifications such as phosphorylation may alter protein stability [44, 45]. Zfp36l1 and Zfp36l2 may be more stable in the hyper-phosphorylated forms than in the hypo-phosphorylated forms. Thus, their protein expression levels are nearly constant even if their mRNA levels decrease after LPS stimulation. However, a recent report showed that Zfp36l2 protein is also down-regulated during LPS treatment [46]. This might be due to a higher dose of LPS and longer stimulation times that were used.

To identify the possible mRNA targets of Zfp36l1 and Zfp36l2 in resting macrophages, we knocked down Zfp36l1 and Zfp36l2 using lentivirus-carrying shRNA. Based on previous reports, the candidate mRNA targets are chosen for analysis by the number of AREs in their 3′UTRs, their association with Ttp, and their mRNA expression profiles, which categorized them as immediate-early genes [27, 30]. We found that *Mkp-1* mRNA increased in Zfp36l1-, Zfp36l2-, and Zfp36l1/Zfp36l2-knockdown cells because of mRNA stabilization (Fig. 3b, c). *Tnfα* is a well-known target of TTP family proteins. Much to our surprise, *Tnfα* mRNA expression decreased in all knockdown cells in the resting state (Fig. 3d). This result may be attributable to the importance of the transcriptional regulation of *Tnfα* mRNA controlled by activation of p38 MAPK [47]. Therefore, the increased Mkp-1 expression in Zfp36l1- and Zfp36l2-knockdown cells repressed the activity of p38 MAPK (Fig. 6a), which down-regulated *Tnfα* mRNA expression. Similarly, *Ccl-2* mRNA expression is also activated by p38 MAPK [48]. This result is consistent with previous reports showing that Mkp-1 overexpression may inactivate Jnk and p38 and thereby inhibit *Tnfα* and *IL-6* expression [39, 49]. However, expression of *Ttp* and *Icam-1* mRNA was not significantly different in control and Zfp36l1- or Zfp36l2-knockdown cells (Fig. 3d). One possible explanation is that expression of their mRNAs is controlled equally at the transcriptional and post-transcriptional levels [50, 51].

The mRNA targets of TTP family proteins are not all the same, although their RNA-binding domains are highly conserved. Ttp-, Zfp36l1-, and Zfp36l2-knockout mice exhibit different phenotypes. Ttp-sensitive targets identified in knockout cells, such as *GM-CSF*, *polo-like-kinase 3*, and *Ier3*, are not regulated in Zfp36l1-knockout cells [22, 25, 52]. On the other hand, ACTH stimulates adrenocortical cells to induce Zfp36l1 and results in down-regulation of *vegf* and *star* mRNAs [53, 54]. *IL-3* mRNA is abnormally stabilized in Zfp36l1-mutated cells [55]. Previously, induced *Mkp-1* mRNA was reported as a Ttp target during the rapid degradation stage after induction [27, 29], and also as a Zfp36l1 and Zfp36l2 target in 3 T3-L1 preadipocytes [26]. In this study, we demonstrated that Zfp36l1 and Zfp36l2 control *Mkp-1* mRNA expression in the resting stage. Thus, *Mkp-1* mRNA stability might be modulated temporally by Ttp family members in LPS-stimulated macrophages. Interestingly, knockdown of either Zfp36l1 or Zfp36l2 affected *Mkp-1* mRNA expression, and no significant additive effect was observed in the double-knockdown cells (Fig. 3b, c). This suggests that there is a functional connection between Zfp36l1 and Zfp36l2.

Figure 4a shows that the interaction between Zfp36l1 and the *Mkp-1* 3′UTR was maintained during LPS stimulation, but the interaction between Zfp36l2 and the *Mkp-1* 3′UTR was decreased. This may be due to the phosphosphorylation of Zfp36l2. Knockdown of Zfp36l1 and Zfp36l2 caused the level of *Mkp-1* mRNA highly increased but its stability not increased dramatically in the early stage of LPS stimulation (Fig. 5b, c). It implied that Zfp36l1 and Zfp36l2 might be inactivated in LPS-stimulated control cells through phosphorylation. This result confirms that the activation of *Mkp-1* mRNA is regulated by Zfp36l1 and Zfp36l2 after LPS stimulation. Associated proteins may change the functions of Zfp36l1 and Zfp36l2. Zfp36l1 and Zfp36l2 may also promote the deadenylation of class II ARE-containing mRNAs [20, 56, 57]. After LPS stimulation, however, Zfp36l1 may be phosphorylated and form a complex with 14-3-3 (Fig. 4b). This complex may repress Zfp36l1 function and thereby stabilize *Mkp-1* mRNA.

## Conclusion

Expression of proinflammatory mediators is suppressed in resting cells of the innate immune system, whereas it is rapidly induced in response to inflammatory stimulation. These suppression and induction require tight controls to maintain the function of immune system. In addition to transcriptional control, these mediator mRNAs are post-transcriptionally regulated [58]. It has been reported that the ribonuclease regnase-1 brakes *IL-6* mRNA expression in resting macrophages, and NF-κB signaling would cause phosphorylation and degradation of regnase-1, thereby releasing this brake [59]. As shown in Fig. 7, our study suggests that Zfp36l1 and Zfp36l2 brake *Mkp-1* mRNA expression in resting macrophages for rapid cellular responses to inflammatory stimulation. Upon stimulation, their mRNA levels were decreased and protein was inactivated by phosphorylation to release *Mkp-1* mRNA blocking.

## Additional files

Additional file 1: Figure S1. The potential AREs located in 3’UTR of *Zfp36l1* and *Zfp36l2* mRNA from sequence NM_007564 2252-2960 and NM_001001806, respectively. The AREs were underlined. Additional file 2: Figure S2. The relative expression levels of *IL-1β*, *IL-6* and *Ccl2* mRNAs in different knockdown cells after LPS stimulation for 15 min. RNA was isolated and performed real-time PCR analysis.

## Abbreviations

Ccl2: Chemokine (C-C motif) ligand2
Caf1: Ccr4-associated factor 1
Ccr4: Carbohydrate catabolism repression 4
ERK: Extracellular signal-regulated kinase
GST: Glutathione S-transferase
IL: Interleukin
JNK: c-Jun amino-terminal kinase
LPS: Lipopolysaccharide
MAPK: Mitogen-activated protein kinase
Mkp1: MAPK phosphatase 1
NOT1: Negative on TATA 1
shRNA: Short hairpin RNA
Tnf: Tumor necrosis factor
TTP: Tristetraprolin
Zfp36l1: Zinc finger protein 36, C3H type-like 1
