# Supplementary material for: Functional regulation of Zfp36l1 and Zfp36l2 in response to lipopolysaccharide in mouse RAW264.7 macrophages
Source: J Inflamm (Lond). 2015 Jul 16;12:42. doi: 10.1186/s12950-015-0088-x (PMC4502546; doi:10.1186/s12950-015-0088-x)
Supplement: Additional file 1: Figure S1. — The potential AREs located in 3’UTR of Zfp36l1 and Zfp36l2 mRNA from sequence NM_007564 2252-2960 and NM_001001806, respectively. The AREs were underlined. [file 12950_2015_88_MOESM1_ESM.docx]

Zfp36l1 3’UTR (NM_007564.5)

2252-GUUGCUUAUCACUGCACAUCAAUAUAAAAAAGC**UUAUUUAA**CUUAUCAAAACG**UAUUUAUU**GCCAAACUAUGCUUUUUUUGUUAAUUUUGUUC**AUAUUUAU**CGGGAUGACAAAUCCAUAGAAUAUAUUCUUUUAUGUUAAAUUAUGAUCUUCAUAUUAAUCUUAAAAUUUUGUGACGUGUCUUUCCUUUUUUCCACAGUUUUAAUAUAUUAUUCUUCAACAACAUUUUUGUAACUUUACACUUUUUUUGG**UUAUUUUAUUUUAAA**AAAAUGAAAAA**UUAAUUUAA**AAAAAUGCAAAAAACUGUUGGA**UUAUUUAUU**UUAGAAAUUCUCCCCUUUGUGUUGGACUGCAAAUUGAGUUUCUUUCUCCUUAGGCCUUUCACAGGUAGGACUGAGAAUGUAUGUAUAAGUUCUGUGACAGUACAGAAGGAAAACCACCAUUUUAUGUAUAGCUUCUAAAAGGGAAAACAAAAAAAGAGAAAAACCCUUUGAAUUCCAUGUGCCCAUCUCAAGACAUUCCGCUCGCAGAUUUGTGGUUCUGGAUUCCAGGUUGGAGUUUUCCAAUGUUGACAUAAACAACUGGCGCACACACAUAAAGAUGAAUGUAAUUAUUAUUCCUCUUGCUGGUCACUACCGUCGCUUUCUAUUUCUCUUUCUUUGUGUG**AAUUUAUU**UAAAAGAAAAAAAAAACUUUUUGUAACGACUAUUUGCAGUUU-2960

Zfp36l2 3’UTR (NM_001001806)

2490-CACCACUGCACCACAACUCAAUAUGAAAAAC**UAUUUAA**C**UUAUUUAUU**AUCUUGUGAAAAGUAUACAUUGAGGAUGUUGUCCAUACUG**UAUUUAU**CGAGUAUGAUGAAAGCAAUAGAUAUAUAUUCUUUUAUUAUGUUAAAUUAUAAUUGCCAUUAUUAAUCGGCAAAAUGUGGAGUGUGUUCUUUUCACAGUAAUAUAUGCCUUUUGUAACUUCACUUGG**UUAUUUUAU**UGUAAAUGAGUAAGAAAAAUCU**UAAUUUAA**GAGAUUGUAUGUAA**UAUUUAUU**UCAUUAAUUUCUUUCCUUGUUUACGUAAAUUUCGAAAGAUCGCAUGAAGAAACCAUGCAA-2831

**Supplemental Figure 1.** The potential AREs located in 3’UTR of *Zfp36l1* and *Zfp36l2* mRNA from sequence NM_007564 2252-2960 and NM_001001806, respectively. The AREs were underlined.
