# Supplementary material for: Functional regulation of Zfp36l1 and Zfp36l2 in response to lipopolysaccharide in mouse RAW264.7 macrophages
Source: J Inflamm (Lond). 2015 Jul 16;12:42. doi: 10.1186/s12950-015-0088-x (PMC4502546; doi:10.1186/s12950-015-0088-x)
Supplement: Additional file 2: Figure S2. — The relative expression levels of IL-1β, IL-6 and Ccl2 mRNAs in different knockdown cells after LPS stimulation for 15 min. RNA was isolated and performed real-time PCR analysis. [file 12950_2015_88_MOESM2_ESM.docx]

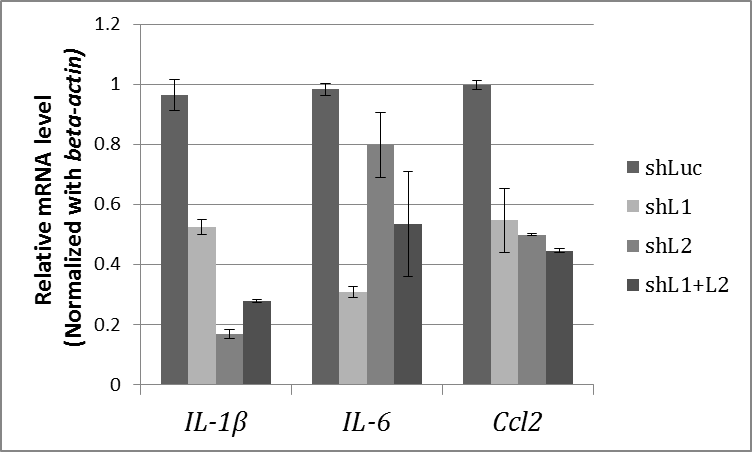


**Supplemental Figure 2.** The relative expression levels of *IL-1β*, *IL-6* and *Ccl2* mRNAs in different knockdown cells after LPS stimulation for 15 min. RNA was isolated and performed real-time PCR analysis.
